# Acquired *Pes Planovalgus* : Current Concepts – “From Adult Acquired *Pes Planovalgus* to Progressive Collapsing Foot Deformity”

**DOI:** 10.1055/s-0044-1793823

**Published:** 2024-12-21

**Authors:** Hugo Bertani Dressler, Kepler Alencar Mendes de Carvalho, Roberto Zambelli, Nacime Salomão Barbachan Mansur, Cesar de Cesar Netto

**Affiliations:** 1 Serviço de Ortopedia e Traumatologia, Rede Mater Dei de Saúde, Belo Horizonte, MG, Brasil; 2 Duke University, Durham, NC, Estados Unidos; 3 Subespecialização em Cirurgia do Pé e Tornozelo, Serviço de Ortopedia e Traumatologia, Rede Mater Dei de Saúde, Belo Horizonte, MG, Brasil; 4 MedStar Orthopedic Institute, MedStar Union Memorial Hospital, Baltimore, MD, Estados Unidos

**Keywords:** adult, flatfoot, foot deformities, posterior tibial tendon dysfunction

## Abstract

The clinical disorder traditionally known as *pes planovalgus due to posterior tibial tendon insufficiency* or *adult-acquired pes planovalgus* has been the subject of several publications over the past two decades. Now, it is understood that the problem does not lie in the posterior tibial tendon per se and may even occur without tendon injury. Studies have brought new concepts and understanding that question the views on this subject, culminating in the replacement of existing classifications with one that is more assertive and discriminative of the potential presentation patterns of the deformity. In addition, a change in the name of the disorder to *progressive collapsing foot deformity* (PCFD) has been proposed.

Regarding surgical treatment, the concept of an *à la carte* approach persists, emphasizing axis realignment through osteotomies, arthrodeses, and soft tissue balancing, which consists of tendon transpositions/repairs and reconstruction of ligament structures, especially the deltoid ligament complex and the spring ligament.

## Introduction

Since the description of the 3 classic stages by Johnson and Strom in 1989, 1 which was modified by Myerson in 1997 2 and, later, by Bluman et al. 3 in 2007, the clinical disorder historically known as *adult acquired pes planovalgus due to insufficiency of the posterior tibial tendon* (PTT) has been the subject of countless publications and questions regarding the role of the PTT in its pathogenesis and the sequence of events that result in the development of this complex, multifactorial, multifocal, and three-dimensional deformity of the foot and ankle.

The literature has dedicated efforts to understanding the disorder better and classifying it in a more reproducible manner to guide treatment and improve its outcomes. The nomenclature used for pes planovalgus deformities, including rupture, dysfunction, or insufficiency of the PTT and acquired pes planovalgus in adults, does not reflect its pathogenesis. It has been recognized that pes planovalgus is not necessarily associated with PTT disruption, it may begin in childhood, and arch flattening is only one aspect of this multiplanar deformity. 4

## Understanding the Deformity

Although PTT dysfunction is widely accepted as a significant contributor to pes planovalgus, there are several other structures involved besides the tendon, 5 which may represent only the tip of the iceberg when considering the degree of medial and plantar soft tissue injury and strain.

Progressive collapsing foot deformity (PCFD) is complex and consists of several components with varying degrees of severity: midfoot abduction deformity, primarily due to lateral deviation at the talonavicular joint; peritalar subluxation, resulting in foot and hindfoot deviation in all three major planes and progressing to subtalar joint eversion; plantar flexion of the talus and forefoot abduction; varus forefoot with the first ray elevated above the fifth metatarsal; and hindfoot valgus. 6

The stages arbitrarily defined as consecutive in the classic classifications can actually occur in a disorganized fashion, without following a spectrum of progression that is necessarily consecutive. Therefore, even if the PTT remains present and has no rupture or insufficiency, deformities can occur, including in the tarsometatarsal joints and the naviculocuneiform joints, potentially with a rupture or attenuation of the plantar fascia, the spring ligament, and the deltoid ligament in any combination. 5 7 Researchers have neglected the significant participation of the spring ligament and its sling effect for decades, and they did not even consider it for classification purposes. 8 It is known that this suspensory effect of the spring ligament evolves with failure, contributing to a collapse worsening.

In summary, the deformities result from an imbalance of bone, muscle, and ligament components acting together.

In the propedeutic evaluation, the following conventional weight-bearing radiographs are required to evaluate patients with PCFD: anteroposterior of the foot and ankle with weight-bearing, mortise, and lateral of the ankle and foot with weight-bearing. 9

**Fig. 1:**
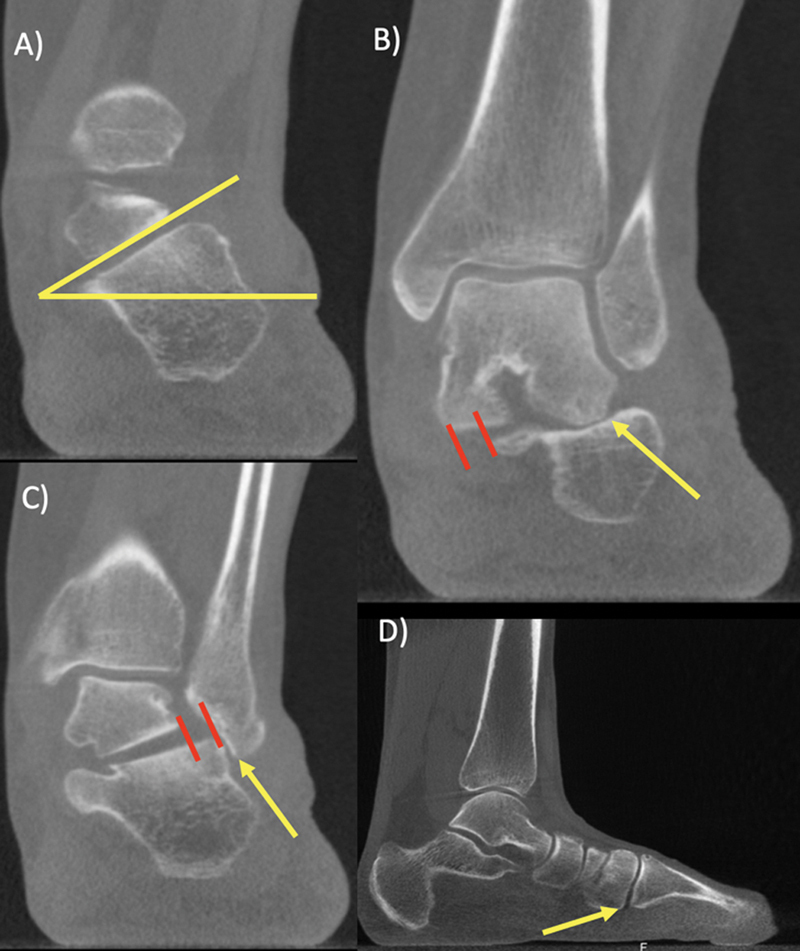
Coronal weight-bearing computed tomography scan demonstrating: ( **A** ) the misalignment of the subtalar joint; ( **B** ) sinus tarsi impingement and medial facet subluxation; and ( **C** ) subfibular impingement and posterior facet subluxation. ( **D** ) Sagittal scan demonstrating instability with the plantar opening of the tarsometatarsal joint.

If available, a hindfoot alignment view is strongly recommended, as is weight-bearing computed tomography (WCT). A WBCT scan from patients with the deformity reveals important findings, such as sinus tarsi impingement, subfibular impingement, increased valgus tilt of the posterior facet of the subtalar joint, and subluxation of the subtalar joint at the posterior and middle facets or both 10 11 ( Fig. 1 ).

**Fig. 2:**
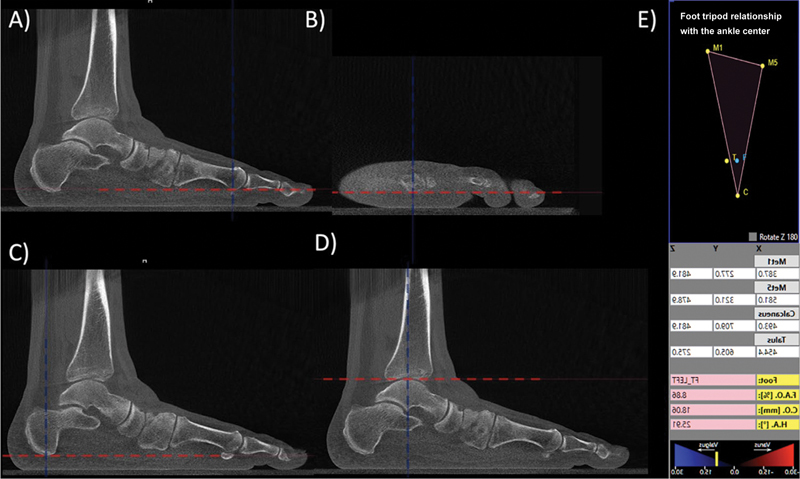
Three-dimensional coordinates to calculate the foot and ankle offset (FAO) weight-bearing computed tomography. ( **A** ) Sagittal section with marking at the lowest point of the head of the first metatarsal; ( **B** ) sagittal section with marking at the lowest point of the head of the fifth metatarsal; ( **C** ) axial section with marking at the lowest point of the calcaneal tuberosity; ( **D** ) sagittal section with marking at the highest point of the talar dome; and ( **E** ) example of a screen display of the software (CubeVue with the TALAS tool, CurveBeam AI, Hatfield, PA, United States) for semiautomatic calculation of the FAO displacement. We evaluated three-dimensional coordinates (x, y, z planes) of weight-bearing computed tomography images for the first (Met1, M1) and fifth (Met5, M5) metatarsals, the calcaneus (C), and the talus (T). These coordinates generated semiautomatic calculations of the foot tripod (triangle) and the ideal (F) and current actual (T) positions of the ankle joint center. In this example, the ankle joint center (T) is medial to the ideal ankle joint center (F), representing a valgus alignment of the hindfoot and an unbalanced tripod.

Although WBCT in Brazil remains available for research purposes alone, not for routine clinical use, it is critical to consider its relevant application to understand deformities. This tool enables the assessment of the relationship between the position of the foot's tripod (weight-bearing points of the first and fifth metatarsal heads and the calcaneal tuberosity) and the center of the ankle joint (the most proximal and central point of the talar dome) and the representation of different components of the three-dimensional deformity in a single measurement, a parameter known as the foot and ankle offset 12 (FAO) ( Fig. 2A–E ). 13

## Updated Nomenclature

Recently, a consensus of experts 14 proposed a new nomenclature and a new classification system, both based on the flexibility, type, and location of the deformities. This group 14 recommended changing the condition's name to PCDF, considering it is a complex and variable three-dimensional deformity. The words *progressive* and *collapsing* convey a better idea of the increasing and evolving nature of the complexity of this multiplanar deformity. These considerations may improve understanding and avoid an underestimation of the disorder, as occurred with the previous terminology.

Since the PTT per se is not the main problem, the new nomenclature does not include it. Furthermore, avoiding the use of *acquired pes planovalgus* as a terminology has been suggested, since many people are born with flatfoot and are never symptomatic; in addition, arch flattening is only one component of a complex, three-dimensional deformity. 7 8 In general, a flatfoot can be a normal finding; however, the factor requiring consideration is a progressive worsening of this deformity or, more precisely, a progressive collapse.

## Updated Classification

The evolution in the understanding of pes planovalgus in adults and the continuous improvement in three-dimensional imaging resulted in the perception of more limitations in the previous classification systems. An ideal classification for any condition should be concise, easy to use, and reproducible, and it should enable universal use and incorporate different deformities, to promote report standardization and guide treatment to achieve optimal outcomes.

The alphanumeric classification system for PCDF consists of 2 sequential stages of flexibility/rigidity of the deformities (stage 1: flexible deformity; and stage 2: rigid deformity) and 5 different deformity classes (A: hindfoot valgus; B: midfoot/forefoot abduction; C: forefoot varus deformity/medial column instability; D: peritalar subluxation/dislocation; and E: ankle valgus deformity), which can occur alone or in any combination. Thus, each deformity class can be flexible or rigid ( Table 1 ). This new classification system has been validated with satisfactory intra- and interexaminer reliability. 7 15 16 17

**Table 1:** Progressive collapsing foot deformity classification 7

Stage 1 (flexible)		Stage 2 (rigid)
Deformity types (classes - isolated or combined)		
	Deformity type/location	Clinical and radiographic findings
Class A	Hindfoot valgus	Hindfoot valgus/increased hindfoot alignment angle and foot and ankle offset
Class B	Midfoot/forefoot abduction	Decreased talar coverage/presence of sinus tarsi impingement
Class C	Forefoot varus/medial column instability	Increased talus–first metatarsal angle/medial plantar tarsometatarsal or naviculocuneiform gap/forefoot varus
Class D	Peritalar subluxation or dislocation	Significant subtalar subluxation/sinus tarsi impingement and subfibular impingement
Class E	Ankle valgus instability	Valgus talar tilt

Therefore, the PCDF classification relies on flexible (stage I) or rigid (stage II) deformities and is further described by the addition of one or more deformities (isolated or combined – classes A to E). A case of rigid hindfoot valgus (2A), flexible and unstable medial column (1C), and forefoot abduction that cannot undergo reduction (2B), for instance, should be reported as A2B2C1, because the examiner will first determine the location of the deformities, and then, whether they are rigid or flexible.

## Treatment

A non-surgical approach should be considered in initial cases and in those with some clinical impediment to the surgery. This treatment modality cannot correct any existing deformity; however, it may improve symptoms and the quality of life of patients. It is possible to use anti-inflammatory medications, insoles, ankle stabilizing orthoses, and rigid shoes with a rocker-bottom or blotting-paper pattern. 18

Considering the deformity spectrum in PCDF, the surgical strategy should be individually evaluated to define the required procedures. Therefore, it is critical to determine the specific existing abnormalities and deformities. This highlights the importance of the new classification, since it considers the type and location (classes) and the flexibility/rigidity of the deformities (stages), reinforcing the *à la carte* nature of the management of this disease.

The general objective of the correction is the realignment of the tripod with the ankle, promoting the correction of the FAO determined by WBCT. 19 Achieving this objective reduces reconstruction failures through calcaneal medialization/lowering osteotomies 6 20 and simple or combined arthrodeses of the hindfoot associated or not with the soft-tissue approach. One must always consider the contracture in the posterior leg muscles, specifically the soleus and gastrocnemius muscles, as a deforming force in hindfoot eversion. This may require lengthening of the gastrocnemius or even of the entire calcaneal tendon and medialization osteotomy of the calcaneus, repositioning it as an inverter. 6

Tendon transfers, such as tenodesis of the peroneus brevis on the peroneus longus, help to reduce the everting moment of the foot and act on the flexion of the first ray by enhancing the action of the peroneus longus. In addition, the approach to the medial structures plays a significant role in tendon retensioning and reconstruction of the deltoid ligament and spring ligament through direct repair and/or reconstruction associated with stabilization tapes. 8 21 22 23 24

One must also consider methods for column realignment, such as lowering and stabilizing the medial column (Cotton, LapiCotton, or both) 25 26 or lateral column alignment (Evans). 23

## Final Considerations

Progressive collapsing deformity of the foot is complex, and it is essential to understand that pes planovalgus is not necessarily a problem requiring intervention but warranting attention and treatment. As the condition progresses and worsens, cases that were initially flexible often become rigid and evolve with clinical and functional worsening.

There is no ideal and clear treatment algorithm to address PCDF deformities, and treatment must be individualized.

In general, surgical intervention for PCDF improves functional performance, but more studies are needed to demonstrate the reliability and durability of these corrections. 27
