# Supplementary material for: Acquired Pes Planovalgus : Current Concepts – “From Adult Acquired Pes Planovalgus to Progressive Collapsing Foot Deformity”
Source: Rev Bras Ortop (Sao Paulo). 2024 Dec 21;59(6):e809–14. doi: 10.1055/s-0044-1793823 (PMC11663050; doi:10.1055/s-0044-1793823)
Supplement: Related texts

# Research article: Pé plano valgo adquirido: Conceitos atuais – “Do pé plano valgo adquirido do adulto à deformidade colapsante progressiva do pé”

**Keywords:** adulto, deformidades do pé, disfunção do tendão tibial posterior, pé chato

## Abstract

O distúrbio clínico tradicionalmente conhecido como *pé plano valgo por insuficiência do tendão tibial posterior* ou *pé plano valgo adquirido do adulto* tem sido objeto de diversas publicações nas últimas duas décadas, e entende-se agora que o problema não está no tendão tibial posterior em si, pois o distúrbio pode ocorrer até mesmo sem a lesão desse tendão. Estudos trouxeram novos conceitos e entendimentos que questionaram o olhar sobre o tema, o que culminou na substituição das classificações existentes por uma que fosse mais assertiva e discriminativa dos possíveis padrões de apresentação da deformidade. Além disso, também propôs-se a mudança da nomenclatura para “deformidade colapsante progressiva do pé” (DCPP; *progressive collapsing foot deformity* , PCFD, em inglês).

Quanto ao tratamento cirúrgico, ainda persiste o conceito de abordagem *à la carte* , com ênfase no realinhamento dos eixos, o que pode ser obtido por meio de osteotomias, artrodeses e equilíbrio de partes moles, que consiste em transposições/reparos tendinosos e reconstrução de estruturas ligamentares, sobretudo do complexo ligamentar deltoide e do ligamento mola.

## Introdução

Desde a descrição, em 1989, dos 3 estágios clássicos de Johnson e Strom, 1 que foi modificada por Myerson em 1997 2 e posteriormente por Bluman et al. 3 em 2007, o distúrbio clínico historicamente conhecido como *pé plano valgo adquirido do adulto por insuficiência do tendão tibial posterior* (TTP) tem sido objeto de incontáveis publicações e questionamentos no que tange ao papel do TTP na patogênese do distúrbio, assim como à sequência de eventos que leva ao desenvolvimento desta deformidade complexa, multifatorial, multifocal e tridimensional do pé e do tornozelo.

A literatura tem dedicado esforços para compreender melhor o distúrbio, além de classificá-lo de maneira mais reprodutível e capaz de guiar o tratamento para melhorar os resultados. A nomenclatura utilizada para deformidades de pé plano, incluindo ruptura, disfunção ou insuficiência do TTP e deformidade de pé plano adquirida em adultos, não reflete a patogênese. Reconheceu-se que a deformidade do pé plano não está necessariamente associada à ruptura do TTP, pode começar na infância e o achatamento do arco é apenas um aspecto desta deformidade multiplanar. 4

## Entendendo a Deformidade

Embora a disfunção do TTP seja amplamente aceita como um fator significativo para o pé plano valgo, há diversas outras estruturas envolvidas além do tendão, 5 que pode representar apenas a ponta do *iceberg* quando o grau de lesão e estiramento de partes moles medial e plantares é levado em consideração.

A deformidade colapsante progressiva do pé (DCPP) é complexa e consiste em vários componentes com nível variável de gravidade: deformidade em abdução do mediopé, principalmente devido ao desvio lateral na articulação talonavicular; subluxação peritalar, que resulta em desvio do pé e do retropé em todos os três planos principais, e evolui com eversão da articulação subtalar; flexão plantar do tálus e abdução do antepé; antepé varo com o primeiro raio elevado acima do quinto metatarso; e retropé valgo. 6

Os estágios arbitrariamente definidos como consecutivos nas classificações clássicas na verdade podem se apresentar de forma desordenada, sem que se respeite um espectro de progressão necessariamente consecutivo. Assim sendo, mesmo que o TTP ainda esteja presente e sem ruptura ou insuficiência, já se pode observar a presença de deformidades, que incluem as das articulações tarsometatarsianas e as das articulações naviculocuneiformes, e que podem estar associadas a uma ruptura ou atenuação da fáscia plantar, do ligamento mola e/ou do ligamento deltoide. 5 7 A participação preponderante do ligamento mola e seu efeito *sling* no colapso foram negligenciados por décadas, inclusive não sendo considerados para fins de classificação. 8 Sabe-se que esse efeito suspensório do ligamento mola evolui com falha, o que contribui para o agravamento do colapso.

Em suma, as deformidades são resultado de um desequilíbrio de componentes ósseos, musculares e ligamentares que atuam conjuntamente.

Na avaliação propedêutica, as seguintes radiografias convencionais com carga são necessárias na avaliação de pacientes com DCPP: anteroposterior do pé e tornozelo com apoio, mortalha, e perfil do tornozelo e pé com apoio. 9

Se disponível, uma visualização do alinhamento do retropé é altamente recomendada, bem como a tomografia computadorizada com carga (TCC). Quando a TCC é obtida em pacientes que apresentam a deformidade, achados importantes a serem identificados são o impacto do seio do tarso, impacto subfibular, aumento da inclinação em valgo da faceta posterior da articulação subtalar e subluxação da articulação subtalar na faceta posterior e/ou média 10 11 ( Fig. 1 ).

**Fig. 1:** Corte coronal de tomografia com carga que demonstra: ( **A** ) o mau alinhamento da subtalar; ( **B** ) o impacto do seio do tarso e a subluxação da faceta média; e ( **C** ) o impacto subfibular e subluxação da faceta posterior. ( **D** ) Corte sagital que demonstra a instabilidade com abertura plantar da articulação tarsometatársica.

Ainda que a TCC no Brasil ainda seja um método disponível apenas para fins de pesquisa, sem disponibilidade para uso clínico de rotina, é importante considerar a relevante aplicação da TCC para a compreensão das deformidades. Por meio desta ferramenta, pode-se avaliar a relação entre a posição do tripé do pé (pontos de sustentação de peso da primeira e quinta cabeças metatarsais, bem como da tuberosidade do calcâneo) e o centro da articulação do tornozelo (ponto mais proximal e central da cúpula do tálus) e representar em uma única medição diferentes componentes da deformidade tridimensional, parâmetro conhecido como *foot and ankle offset* 12 (FAO) ( Fig. 2A–E ). 13

**Fig. 2:** Coordenadas tridimensionais para o cálculo do *foot and ankle offset* (FAO) na tomografia com carga. ( **A** ) Corte sagital com marcação no ponto mais inferior da cabeça do 1° metatarso; ( **B** ) corte sagital com marcação no ponto mais inferior da cabeça do 5° metatarso; ( **C** ) corte axial com marcação no ponto mais inferior da tuberosidade do calcâneo; e ( **D** ) corte sagital com marcação no ponto mais alto do domo talar. e ( **E** ) Exemplo de visualização de tela do programa (CubeVue com a ferramenta TALAS, CurveBeam AI, Hatfield, PA, Estados Unidos) usado para o cálculo semiautomático do deslocamento do FAO. Coordenadas tridimensionais (planos x, y, z) de imagens de tomografia computadorizada com carga foram avaliadas para o primeiro (Met1, M1) e o quinto (Met5, M5) metatarsos, o calcâneo (C) e o tálus (T). Essas coordenadas são usadas para gerar cálculos semiautomáticos do tripé do pé (triângulo), bem como as posições ideal (F) e real atual (T) do centro da articulação do tornozelo. Neste exemplo específico, o centro da articulação do tornozelo (T) está medial ao centro ideal da articulação do tornozelo (F), o que representa um alinhamento em valgo do retropé e um tripé desequilibrado.

## Nomenclatura Atualizada

Recentemente, um consenso de especialistas 14 propôs uma nova nomenclatura e um novo sistema de classificação, ambos baseados na flexibilidade, no tipo e na localização das deformidades. Esse grupo 14 recomendou a mudança de nomenclatura dessa patologia para DCPP, considerada uma deformidade tridimensional complexa e variável. As palavras *progressiva* e *colapsante* dão uma ideia melhor da natureza crescente e evolutiva da complexidade desta deformidade multiplanar. Essas considerações podem melhorar o entendimento e evitam uma subestimação do distúrbio, conforme ocorria com a terminologia anterior.

Uma vez que o TTP em si não é o principal problema, o nome do tendão não foi incluído na nova nomenclatura da doença. Além disso, sugere-se não utilizar *pé plano adquirido* como terminologia, pois muitas pessoas nascem com pé plano e nunca são sintomáticas, e o achatamento do arco é apenas um dos componentes de uma deformidade complexa e tridimensional. 7 8 De maneira geral, ter um pé plano pode ser um achado normal; no entanto, o fator que deve ser considerado é uma piora progressiva desta deformidade, ou, mais precisamente, um colapso progressivo.

## Classificação Atualizada

A evolução na compreensão da patologia da deformidade do pé plano em adultos e a melhoria contínua nas imagens tridimensionais levaram à percepção de mais limitações nos sistemas de classificação anteriores. Uma classificação ideal para qualquer patologia deve ser concisa, fácil de usar, reprodutível e passível de ser usada universalmente, e deve incorporar diferentes deformidades, o que permite a padronização dos relatórios e a orientação do tratamento para alcançar resultados ideais.

O sistema alfanumérico de classificação da DCPP consiste em 2 estágios sequenciais de flexibilidade/rigidez das deformidades (estágio 1: deformidade flexível; e estágio 2: deformidade rígida) e 5 diferentes classes de deformidade (A: retropé valgo; B: abdução do mediopé/antepé; C: deformidade em varo do antepé/instabilidade da coluna medial; D: subluxação/luxação peritalar; e E: deformidade em valgo do tornozelo), que podem ocorrer isoladamente ou em qualquer combinação. Desta forma, cada classe de deformidade pode ser flexível ou rígida ( Tabela 1 ). Esse novo sistema de classificação já foi validado, com satisfatória confiabilidade intra e interexaminador. 7 15 16 17

**Tabela 1:** Classificação da deformidade colapsante progressiva do pé 7

Estágio 1 (flexível)		Estágio 2 (rígido)
Tipos de deformidade (classes – isoladas ou combinadas)		
	Deformidade: tipo /localização	Achados clínicos e radiográficos
Classe A	Retropé valgo	Retropé valgo/Aumento do ângulo de alinhamento do retropé e do *foot and ankle offset*
Classe B	Abdução do mediopé/antepé	Diminuição da cobertura talar/Presença de impacto no seio do tarso
Classe C	Varo do antepé/instabilidade da coluna medial	Aumento do ângulo entre o talo e o 1° metatarso/ *gap* plantar medial tarsometatarsal ou naviculocuneiforme/varo do antepé
Classe D	Subluxação ou luxação peritalar	Significativa subluxação subtalar/impacto do seio do tarso e impacto subfibular
Classe E	Instabilidade em valgo do tornozelo	*Tilt* talar em valgo

Assim sendo, a classificação da DCPP é baseada em deformidades que são flexíveis (estágio I) ou rígidas (estágio II) e descritas posteriormente pelo acréscimo de uma ou mais deformidades (isoladas ou combinadas: classes A a E). Um caso com retropé valgo rígido (2A), coluna medial flexível e instável (1C) e abdução do antepé que não pode ser reduzida (2B), por exemplo, deve ser relatado como A2B2C1, pois o examinador irá primeiro determinar onde estão as deformidades e, em seguida, determinar se as deformidades são rígidas ou flexíveis.

## Tratamento

A abordagem não cirúrgica deve ser considerada nos casos iniciais e naqueles que eventualmente possam apresentar algum impedimento clínico para o procedimento cirúrgico. Essa modalidade de tratamento não é capaz de corrigir qualquer deformidade já instalada, mas pode ser suficiente para proporcionar alguma melhora dos sintomas e da qualidade de vida do paciente. Pode-se utilizar medicações anti-inflamatórias e prescrição de palmilhas, órteses estabilizadoras de tornozelo, bem como o uso de calçados rígidos e padrão *rocker bottom ou* mata-borrão. 18

Considerando a ampla variedade de deformidades que podem estar presentes nos casos de DCPP, é possível compreender que a estratégia cirúrgica deverá ser individualmente avaliada para se definir quais procedimentos serão necessários. Assim sendo, é importante definir especificamente quais alterações e deformidades estão presentes e, nesse sentido, nota-se a importância da nova classificação, que considera o tipo e a localização (classes), assim como a flexibilidade/rigidez das deformidades (estágios), o que reforça o caráter *à la carte* do manejo da doença.

O objetivo geral da correção deve ser o realinhamento do tripé com o tornozelo, para promover a correção do FAO, determinado pela TCC. 19 O alcance desse objetivo diminui as falhas na reconstrução, mediante osteotomias de medialização/abaixamento do calcâneo, 6 20 artrodeses simples ou conjugadas do retropé, associadas ou não a abordagem de partes moles. A contratura na musculatura posterior da perna, mais especificamente do músculo solear e do gastrocnêmio como força deformante em eversão do retropé, deve ser sempre considerada. Neste sentido, o alongamento do gastrocnêmio ou até mesmo de todo o tendão calcâneo podem se fazer necessários, assim como a osteotomia de medialização do calcâneo, que reposiciona o tendão de Aquiles como um inversor. 6

As transferências tendíneas, tais como a tenodese do fibular curto no fibular longo, contribuem para diminuir o momento eversor do pé e atuam na flexão do primeiro raio ao se potencializar a ação do fibular longo. Além disso, a abordagem das estruturas mediais tem papel preponderante, seja no retensionamento tendíneo, bem como na reconstrução do ligamento deltoide e do ligamento mola, por meio de reparo direito e/ou reconstrução associada a fitas de estabilização. 8 21 22 23 24

Métodos de realinhamento das colunas devem ser considerados, tais como o abaixamento e a estabilização da coluna medial (Cotton e/ou LapiCotton), 25 26 bem como o alinhamento da coluna lateral (Evans). 23

## Considerações Finais

A DCPP é complexa, e é fundamental a compreensão de que ter um pé plano valgo não necessariamente é um problema que demande intervenção, mas ter um pé plano valgo progressivo merece atenção e abordagem. Com a progressão e o agravamento do quadro, os casos inicialmente flexíveis tendem a se tornar rígidos e evoluir com piora clínica e funcional.

Não existe um algoritmo de tratamento ideal e claro que aborde as deformidades na DCPP, e o tratamento deve ser individualizado.

No geral, a intervenção cirúrgica para DCPP leva claramente a melhorias no desempenho funcional, mas são necessários mais estudos para demonstrar a confiabilidade e a durabilidade destas correções. 27
